# Size matters: How safety climate and downstream outcomes vary by fire department organization type

**DOI:** 10.1186/s40621-022-00373-x

**Published:** 2022-03-23

**Authors:** Ashley M. Geczik, Jin Lee, Andrea L. Davis, Joseph A. Allen, Jennifer A. Taylor

**Affiliations:** 1 Department of Environmental and Occupational Health, Dornsife School of Public Health at Drexel University, Philadelphia, PA USA; 2 Department of Psychological Sciences, Kansas State University, Manhattan, KS USA; 3 Department of Family and Preventative Medicine, University of Utah, Salt Lake City, UT USA

**Keywords:** Safety climate, Fire service, Organizational culture, Safety and organizational outcomes, Injury prevention, Well-being outcomes

## Abstract

**Background:**

Safety climate is an upstream predictor of safety behaviors (e.g., safety compliance), organizational outcomes (e.g., burnout, engagement), and safety outcomes (e.g., injuries). The Fire Service Organizational Culture of Safety (FOCUS) survey, which was psychometrically validated, measures the industry-specific safety climate of the US fire and rescue service. It is expressed by two factors, Management Commitment to Safety and Supervisor Support for Safety.

**Methods:**

The FOCUS beta-test included a random sample of 132 fire departments stratified by Federal Emergency Management Agency region and organization type (career, combination, volunteer). We conducted descriptive analysis with the responses from 8414 firefighters nested within 611 stations in 125 fire departments. We reported descriptive statistics to assess the distribution of all continuous [mean ± standard deviation (SD)] and categorical variables (counts, percentages) stratified by organization type. Regression analyses were conducted to investigate the associations between safety climate, safety behaviors, organizational outcomes, and safety outcomes stratified by organization type.

**Results:**

The mean age of the analytic sample was 40.2 years, and the mean years of experience was 16.1 years. This sample included 53.6% career, 27.2% combination (career and volunteer), and 19.2% volunteer fire departments. The mean Management Commitment score was 71.4 (SD =  ± 10.4), and the mean Supervisor Support score was 81.7 (± 5.2). The mean Management Commitment scores were 67.1 (± 8.4), 72.2 (± 10.7), and 82.1 (± 6.1), respectively, for career, combination, and volunteer fire departments. The mean Supervisor Support scores were not notably different by organization type. Regression analyses generally supported the beneficial role of safety climate, while suggesting organization type as a potential effect modifier. Specifically, we observed a more negative association between Management Commitment as departments became more career.

**Conclusions:**

Analysis of nationally representative data from the US fire and rescue service indicates safety climate is positively associated with safety behavior, organizational outcomes, and safety outcomes reflecting employee well-being. The findings also suggest that this association varies by organization type. In fact, a dose–response relationship was observed, with Management Commitment to safety lowest among career departments. Thus, our results suggest that it is not just being busy that decreases Management Commitment.

## Background

Safety climate is defined as the shared perceptions of employees regarding their organization’s safety policies, procedures, and practices and how different kinds of behavior that are supported and rewarded by leadership within the organization (Zohar 1980). Safety climate has been identified as one of the most pronounced upstream predictors of safety behaviors (e.g., safety compliance), and safety outcomes (e.g., injuries), as well as organizational outcomes (e.g., burnout, engagement) (Christian et al. 2009; Huang et al. 2016). Understanding an organization’s safety climate can allow for organization-level changes that can improve the downstream effects resulting in improved safety behaviors, a reduction in injuries, and improved perceptions of organizational outcomes.

The Fire Service Organizational Culture of Safety (FOCUS) survey measures the industry specific safety climate of the US fire and rescue service (Davis et al. 2020; Taylor et al. 2019). In 2018, it was estimated that the US fire service was comprised of approximately 1.1 million firefighters, of which 745,000 (67%) were volunteer employees and 370,000 (33%) were career employees (Evarts and Stein 2020). According to the National Fire Incident Reporting System, in 2017, 64% of the 26,880,800 calls that fire departments received required emergency medical services (EMS) or rescue services response (United States Fire Administration 2019). The purpose of FOCUS is to provide objective data for fire department decision making to prevent injuries and increase well-being through measurement, monitoring, and management of safety climate. The first wave of FOCUS has been referred to as the FOCUS beta-test survey (Taylor et al. 2019). The FOCUS beta-test survey obtained information on fire department organization type (“organization type”) for all departments. Organization type is categorized as career, combination (career and volunteer), and volunteer fire departments. FOCUS has been previously psychometrically validated using a geographically stratified random sample of 130 fire departments including 615 stations and 8575 firefighters (Taylor et al. 2019). In brief, a 14-item multi-level measure of industry specific safety climate for the fire service was developed (Taylor et al. 2019). The dissemination of FOCUS survey results has been previously described (Davis et al. 2020). Briefly, findings and implications of the FOCUS beta-test survey were shared with the participating departments in a 7-page report comparing benchmarks of their data to other fire departments. Each participating department is provided with their department-specific report, which includes their overall FOCUS score, scores for Management Commitment to Safety (Management Commitment), and Supervisor Support for Safety (Supervisor Support) at the department level. Then at the station-level their FOCUS score is reported, along with burnout on EMS and fire, engagement on EMS and fire, and job satisfaction.

In line with the previous studies regarding the common attributes of safety climate (Flin et al. 2000; Yule et al. 2007), the FOCUS survey operationalized safety climate by two factors, Management Commitment and Supervisor Support (Taylor et al. 2019). Our current work is based upon the safety climate to safety outcomes causal pathway. Our previous work has informed us that safety climate precedes safety behaviors followed by organizational outcomes followed by safety outcomes. Organizational outcomes relate to burnout, engagement, and job satisfaction (Davis et al. 2020; Taylor et al. 2019). Safety outcomes include occupational injuries and near misses (Davis et al. 2020; Taylor et al. 2019). Our previous work informs us that burnout is typically higher on emergency medical services (EMS) runs versus on fire runs and that engagement is typically higher on fire runs versus on EMS runs (Davis et al. 2020; Taylor et al. 2019). Also, notable variations in burnout and engagement were found across fire departments (Davis et al. 2020). One important question that needs to be answered is whether these differences in safety climate and its potential outcomes are systematic and which organizational contexts or structural elements are associated with the differences.

Prior investigations into the role of organizational safety climate within the fire service evaluated different outcome measures among career firefighters (Smith 2020; Smith et al. 2019, 2020). One study examined the impact of affective organizational commitment, defined as a determinant of one’s dedication to their organization, on firefighter safety (Smith 2020). It found that affective organizational commitment is associated with positive safety behavior outcomes among career firefighters (Smith 2020). Another study developed and validated a multi-level safety climate measure for the fire service by surveying two metropolitan fire departments (Smith et al. 2019). Building upon this research, additional work in these departments was conducted to examine the association between stress and burnout with safety behaviors of career firefighters (Smith et al. 2020). The authors concluded that burnout negatively affects the safety behavior outcomes of compliance and safety citizenship among career firefighters (Smith et al. 2020). However, these findings might be specific to the unique governance structures or leadership approaches to career fire departments only and not readily generalizable to volunteer or combination departments.

In the current study, we evaluated the descriptive characteristics that differed between mean FOCUS scores of Management Commitment and Supervisor Support among the FOCUS beta-test respondents. Our primary research aim was to examine how the individual demographic and department-level data differed by safety climate among participating fire departments. Our secondary aim was to investigate the differences of safety climate scores by fire department organization type. Our tertiary aim was to investigate the association between self-reported injury status and safety climate by organization type. This study describes the FOCUS beta-test survey data with particular emphasis on responding to queries, such as the impact of busyness, raised by participating fire departments for the effective promotion of safety climate.

## Methods

### Population

The FOCUS beta-test survey was a random sample of career, combination, and volunteer fire departments (*n* = 132), which was geographically stratified across the 10 US Federal Emergency Management Agency (FEMA) regions. Its design, recruitment, and psychometric properties have been described previously (Davis et al. 2020; Taylor et al. 2019). In brief, we have data collected from three levels: individual, station, and fire department. Fire departments were encouraged to achieve at least a 60% response rate, at the station level, when participating in the FOCUS survey beta-test. The FOCUS beta-test included 10,073 individuals nested within 757 stations in 132 fire departments. Our baseline analytic sample was that of the FOCUS beta-test psychometric analysis, which was comprised of 8575 individuals nested within 615 stations in 130 fire departments (Taylor et al. 2019). Our exclusionary criteria for analysis were as follows: stations that did not have an EMS component (*n* = 49 individuals, *n* = 4 stations), respondents that did not complete any of the demographic questions (*n* = 83 individuals), respondents that had missing safety climate scores (*n* = 29 individuals, *n* = 3 departments), and departments that did not complete their supplementary department-level demographic survey (*n* = 2). This resulted in an analytic sample of 8414 individuals nested within 611 stations in 125 fire departments. Our analytic sample had an average 66% response rate to the FOCUS beta-test survey at the department level.

### Descriptive variables

Descriptive and demographic characteristics were obtained from the individual respondents of the FOCUS beta-test survey. In the survey, individuals were asked to “select all that apply” when identifying their rank and race and ethnicity. In the event that an individual selected more than one response they were categorized as “more than one selected” for rank and “more than one race” for race and ethnicity. We reduced the number of categories for rank due to small sample sizes when stratified by organization type and injury status. For rank we created a three-level categorical variable (non-officer, officer, leadership), referred to as officer status. The non-officer category included individuals that identified as a firefighter, emergency medical technician (EMT), or paramedic. The officer category included individuals that identified as a lieutenant or captain. The leadership category included individuals that identified as a battalion chief, chief, or commissioner. Individuals that had selected more than one rank were classified based on the highest level of rank they selected.

Additionally, select descriptive characteristics were collected from each fire department to obtain the demographic make-up of the department. For Insurance Services Office (ISO) rating, some departments reported two scores since rural and urban areas have a different scoring, typically. In the event that two scores were reported by a fire department the poorer score was used for the descriptive statistics. Of note, the roster size, annual call volume, and population served variables are naturally continuous.

### Safety Climate

The two FOCUS safety climate scores we examined were mean Management Commitment scores and mean Supervisor Support scores. FOCUS Management Commitment is defined as firefighter perceptions of how leadership values and supports safety within the organization and has been conceptualized as a department-level safety climate dimension (Taylor et al. 2019). FOCUS Supervisor Support is defined as department members’ perceptions of the commitment to safety by their direct supervisor (e.g., captain, lieutenant) in how they value and support safety within their crew and has been conceptualized as a station-level safety climate dimension (Taylor et al. 2019). Scores for each of these domains were measured through self-reported responses for seven items on a 5-point Likert scale (strongly disagree, disagree, neither agree nor disagree, agree, and strongly agree) from the FOCUS survey instrument. Mean scores were converted to a 100-point scale for interpretability by the fire service. As an example, if the mean score was 3.7, it was converted to 74.0 by multiplying 3.7 by 20.

### Safety compliance behavior

Safety compliance behavior, referred to as safety compliance, is defined as the degree of accordance by a member to established safety protocols, processes, and standards by members for fire-based response and was assessed using an adaptation from the Vulnerability Assessment Project, which has been used by fire department leadership to evaluate and assess risks associated with exposures, injuries, and line of duty deaths (National Fallen Firefighters Foundation 2014). Responses were obtained using a 5-point Likert scale (strongly disagree, disagree, neither agree nor disagree, agree, and strongly agree) through self-reported responses by individuals. Mean scores were converted to a 100-point scale for interpretability.

### Organizational outcomes

Organizational outcomes representing firefighters’ well-being at work domain (burnout on EMS and fire runs, engagement on EMS and fire runs, and job satisfaction) were obtained using a 5-point Likert scale (strongly disagree, disagree, neither agree nor disagree, agree, and strongly agree). This information was obtained through self-reported responses among individual respondents. Each survey included questions regarding burnout on EMS and fire runs and engagement on EMS and fire runs. Thus, the same individual completed the corresponding questions thinking about their perceived burnout and engagement on EMS runs versus their fire runs. Mean scores were converted to a 100-point scale for interpretability. These organizational outcomes have been previously defined and their measures were validated (Taylor et al. 2019). Burnout was derived from Maslach’s Burnout Inventory and is defined as emotional exhaustion and depersonalization due to the chronic strain of an individual’s work (Maslach and Jackson 1981). It should be noted that burnout scores for FOCUS are interpreted as more positive the lower the score is (Davis et al. 2020). For example, the lower a department’s burnout score, the less burnout those members reported experiencing. Engagement is defined as the vigor, absorption, and dedication of one’s work-related state and was assessed using a 6-item scale that measured employee engagement (Schaufeli et al. 2002). Job satisfaction is defined as the level of positivity about work, sometimes referred to as morale and was assessed using an adaptation from the Safety Attitudes Questionnaire’s subscale on job satisfaction (Sexton et al. 2006).

### Statistical analysis

We reported descriptive statistics to assess the distribution of all continuous (mean ± standard deviation (SD), range) and categorical variables (counts, percentages) for the analytic sample stratified by organization type. Descriptive and demographic variables were collected at the individual level and department level.

Pearson correlation matrixes were run for continuous variables to investigate correlations between covariates and mean safety climate scores at the department level as well as at the individual level. Linear regression models were used to estimate the relationship between organizational outcomes and the two dimensions of FOCUS safety climate: Management Commitment and Supervisor Support. The linear regression models were adjusted for roster size, annual call volume, and population served. These size variables were recategorized based on quartiles for each corresponding organization type.

Logistic regression was used to estimate the odds of self-reported injury 12 months prior to completing FOCUS associated with safety climate scores (Management Commitment, Supervisor Support). Multilevel logistic regression was used to calculate the beta estimates that were then exponentiated to estimate the odds ratios (OR) and 95% confidence intervals (CI). For the multilevel models, individual-level injury status (yes/no) was regressed on department-level Management Commitment or Supervisor Support scores, while random effects were specified at the department and station levels. These models were adjusted for age, years of experience, sex (male, female), and officer status (non-officer, officer, leadership). Due to missing data, individuals missing injury status (*n* = 398), sex (*n* = 224), and officer status (*n* = 103) were excluded from these models. Statistical significance was set to < 0.05 for all analyses.

The protocol received Institutional Review Board approval from Drexel University. All statistical analyses were conducted using SAS 9.4 (Cary, North Carolina).

## Results

**Fig. 1:**
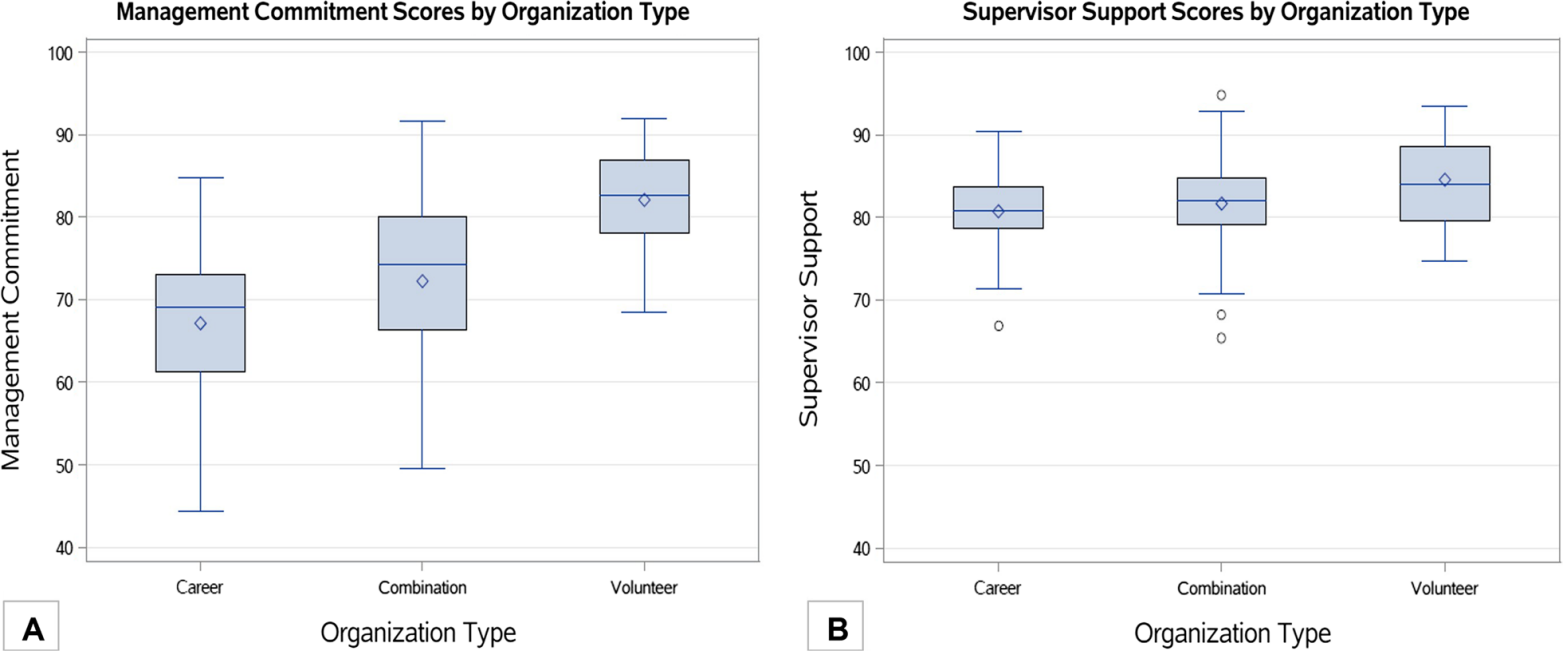
Box and whisker plots comparing Management Commitment and Supervisor Support scores by organization type. **A** Comparison of mean Management Commitment scores by organization type (career, combination, volunteer); **B** Comparison of mean Supervisor Support scores by organization type (career, combination, volunteer)

Our sample included a total of 125 fire departments comprised of three organization types: 67 (53.6%) career, 34 (27.2%) combination (career and volunteer), and 24 (19.2%) volunteer fire departments. We report the descriptive characteristics of the beta-test respondents for the total population and stratified by organization type in Table 1. In total, 1406 individuals (16.7%) reported that they had experienced an injury in the 12 months prior to completing the survey. There were differences in organizational outcomes when participants were thinking about their work on an EMS run versus a fire run, most notably for engagement. The overall mean engagement on EMS was 70.9 (41.7–88.2) versus 80.3 (60.7–91.8) on fire runs. We did not observe notable differences by organization type. The mean Management Commitment score was 71.4 (SD ± 10.4) and the mean Supervisor Support score was 81.7 (± 5.2) for all departments. The mean Management Commitment score varied between organizational types, indicating a dose–response relationship (Fig. 1A). Which was not observed for Supervisor Support (Fig. 1B).

**Table 1:** Descriptive characteristics of FOCUS beta-test analytic sample stratified by fire department organization type

Individual-level characteristics	Total population		Career department		Combination department		Volunteer department	
	*n* = 8414		*n* = 6900		*n* = 1132		*n* = 382	
	Mean ± SD	Min–max	Mean ± SD	Min–max	Mean ± SD	Min–max	Mean ± SD	Min–max
Age	40.2 ± 4.8	23.6–59.0	40.5 ± 4.6	23.6–56.2	39.0 ± 5.2	27.6–59.0	39.1 ± 6.7	28.7–57.3
Years of experience	16.1 ± 4.5	3.4–34.1	16.0 ± 4.3	5.1–31.9	15.7 ± 4.7	3.4–31.1	17.4 ± 6.4	7.4–34.1

Individual-level characteristics	Total population		Career department		Combination department		Volunteer department	
	*n*	%	*n*	%	*n*	%	*n*	%
Sex								
Male	7665	91.1	6312	91.5	1027	90.7	326	85.3
Female	470	5.6	371	5.4	55	4.9	44	11.5
Missing	279	3.3	217	3.1	50	4.4	12	3.1
Rank								
Firefighter	1929	22.9	1454	21.1	271	23.9	204	53.4
Paramedic	157	1.9	149	2.2	8	0.7	0	0.0
EMT	54	0.6	40	0.6	10	0.9	4	1.0
Lieutenant	718	8.5	654	9.5	49	4.3	15	3.9
Captain	922	11.0	779	11.3	128	11.3	15	3.9
Battalion Chief	311	3.7	260	3.8	42	3.7	9	2.4
Chief/Commissioner	68	0.8	31	0.4	21	1.9	16	4.2
More than one selected	4082	48.5	3396	49.2	581	51.3	105	27.5
Missing	173	2.1	137	2.0	22	1.9	14	3.7
Officer status^a^								
Non-officer	5476	65.1	4406	63.9	798	70.5	272	71.2
Officer	2331	27.7	2034	29.5	240	21.2	57	14.9
Leadership	434	5.2	323	4.7	72	6.4	39	10.2
Missing	173	2.1	137	2.0	22	1.9	14	3.7
Race and Ethnicity								
White	6020	71.5	4754	68.9	917	81.0	349	91.4
Black or African-American	432	5.1	408	5.9	24	2.1	0	0.0
Hispanic	743	8.8	708	10.3	29	2.6	6	1.6
Asian/Native Hawaiian/Pacific Islander	72	0.9	63	0.9	9	0.8	0	0.0
American Indian/American Native	69	0.8	61	0.9	6	0.5	2	0.5
More than one race	379	4.5	332	4.8	40	3.5	7	1.8
Other	265	3.1	232	3.4	31	2.7	2	0.5
Missing	434	5.2	342	5.0	76	6.7	16	4.2
Education								
Less than high school	23	0.3	13	0.2	2	0.2	8	2.1
High school or equivalent	2694	32.0	2189	31.7	354	31.3	151	39.5
Undergraduate degree	3452	41.0	2966	43.0	406	35.9	80	20.9
Graduate degree	708	8.4	595	8.6	90	8.0	23	6.0
Missing	1537	18.3	1137	16.5	280	24.7	120	31.4
Injury last 12 months								
No	6610	78.6	5396	78.2	883	78.0	331	86.6
Yes	1406	16.7	1197	17.3	191	16.9	18	4.7
Missing	398	4.7	307	4.4	58	5.1	33	8.6

Fire department characteristics	Total departments		Career department		Combination department		Volunteer department	
	*n* = 125		*n* = 67		*n* = 34		*n* = 24	
	Mean ± SD	Min–max	Mean ± SD	Min–max	Mean ± SD	Min–max	Mean ± SD	Min–max
Percent EMS runs	64.6 ± 22.2	0.0–98.0	69.4 ± 13.0	20.0–98.0	72.9 ± 10.6	41.2–92.0	64.1 ± 34.8	0.0–80.0
Percent fire runs	29.7 ± 25.8	1.9–100.0	22.2 ± 16.3	2.0–80.0	23.6 ± 17.2	1.9–88.0	35.7 ± 34.5	14.0–100.0
Injury rate	13.7 ± 14.7	0.0–70.2	19.5 ± 15.5	0.0–70.2	10.0 ± 12.3	0.0–47.1	2.6 ± 3.7	0.0–13.3
Burnout on EMS runs	45.4 ± 5.3	35.7–59.6	46.6 ± 4.6	36.2–59.6	44.0 ± 5.4	35.7–54.3	46.3 ± 6.6	37.1–58.3
Burnout on fire runs	42.1 ± 3.6	33.3–54.1	42.4 ± 3.2	33.3–51.4	41.1 ± 4.0	34.7–51.2	42.6 ± 4.1	34.2–54.1
Engagement on EMS runs	70.9 ± 7.2	41.7–88.2	69.4 ± 4.5	58.5–82.0	73.0 ± 6.8	58.1–87.8	72.2 ± 11.8	41.7–88.2
Engagement on fire runs	80.3 ± 4.4	60.7–91.8	79.8 ± 3.7	69.0–89.0	81.3 ± 4.3	71.9–89.5	80.2 ± 6.1	60.7–91.8
Job satisfaction	78.3 ± 7.1	61.5–96.0	76.1 ± 6.5	61.5–95.0	78.8 ± 7.3	65.9–95.0	83.8 ± 5.2	74.4–96.0
Safety compliance	80.6 ± 6.7	58.6–94.8	80.0 ± 6.9	58.6–93.8	81.8 ± 6.1	67.8–94.8	80.2 ± 7.2	62.8–92.4
Management Commitment	71.4 ± 10.4	44.3–92.0	67.1 ± 8.4	44.3–84.8	72.2 ± 10.7	49.5–91.7	82.1 ± 6.1	68.5–92.0
Supervisor Support	81.7 ± 5.2	65.5–94.9	80.7 ± 4.1	66.9–90.4	81.7 ± 6.2	65.5–94.9	84.6 ± 5.6	74.8–93.4

Fire department characteristics	Total departments		Career department		Combination department		Volunteer department	
	*n*	%	*n*	%	*n*	%	*n*	%
Roster size								
0–24	25	20.0	10	14.9	7	20.6	8	33.3
25–49	36	28.8	10	14.9	14	41.2	12	50.0
50–99	32	25.6	19	28.4	9	26.5	4	16.7
100+	32	25.6	28	41.8	4	11.8	0	0.0
Annual number of calls								
0–499	21	16.8	0	0.0	6	17.6	15	62.5
500–999	12	9.6	0	0.0	3	8.8	9	37.5
1000–4999	40	32.0	26	38.8	14	41.2	0	0.0
5000–9999	19	15.2	13	19.4	6	17.6	0	0.0
10,000+	28	22.4	24	35.8	4	11.8	0	0.0
Missing	5	4.0	4	6.0	1	2.9	0	0.0
Population served								
0–4999	16	12.8	1	1.5	5	14.7	10	41.7
5000–9999	14	11.2	5	7.5	4	11.8	5	20.8
10,000–24,999	28	22.4	10	14.9	12	35.3	6	25.0
25,000–49,999	21	16.8	13	19.4	6	17.6	2	8.3
50,000–99,999	19	15.2	16	23.9	3	8.8	0	0.0
100,000+	25	20.0	21	31.3	4	11.8	0	0.0
Missing	2	1.6	1	1.5	0	0.0	1	4.2
FEMA region								
1	12	9.6	6	9.0	3	8.8	3	12.5
2	12	9.6	7	10.4	0	0.0	5	20.8
3	14	11.2	5	7.5	3	8.8	6	25.0
4	11	8.8	9	13.4	2	5.9	0	0.0
5	14	11.2	5	7.5	6	17.6	3	12.5
6	11	8.8	7	10.4	3	8.8	1	4.2
7	9	7.2	6	9.0	1	2.9	2	8.3
8	11	8.8	6	9.0	3	8.8	2	8.3
9	16	12.8	11	16.4	4	11.8	1	4.2
10	15	12.0	5	7.5	9	26.5	1	4.2
CPSE accreditation								
No	103	82.4	52	77.6	29	85.3	22	91.7
Yes	13	10.4	10	14.9	3	8.8	0	0.0
Missing	9	7.2	5	7.5	2	5.9	2	8.3
ISO rating								
High (1, 2, 3)	54	43.2	43	64.2	8	23.5	3	12.5
Medium (4, 5, 6)	49	39.2	16	23.9	19	55.9	14	58.3
Low (7, 8, 9, 10)	13	10.4	1	1.5	6	17.6	6	25.0
Missing	9	7.2	7	10.4	1	2.9	1	4.2

^a^If more than one rank was selected, the highest level of rank was designated for this categorization

**Fig. 2:**
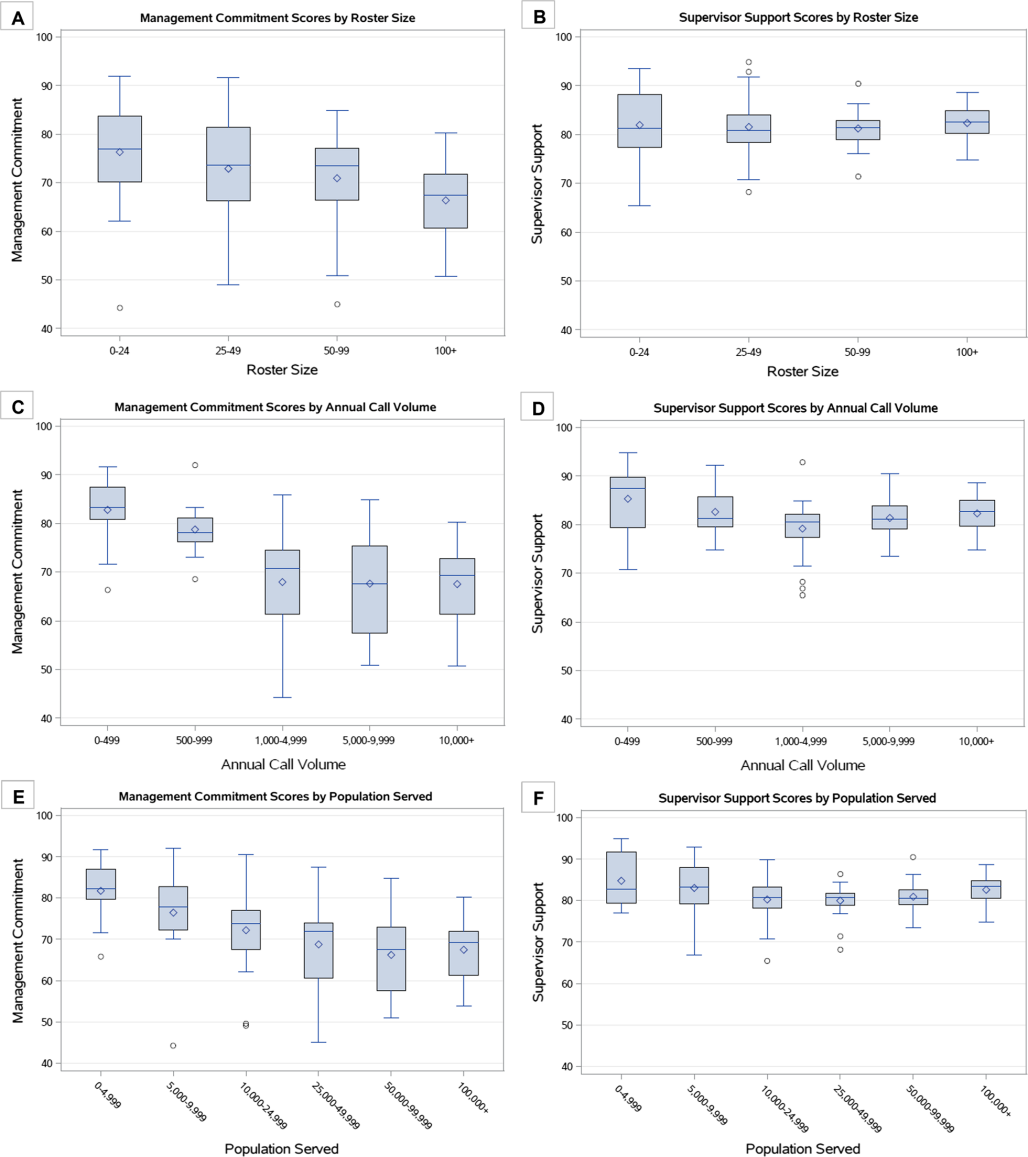
Box and whisker plots comparing Management Commitment and Supervisor Support scores by size variables. **A** Comparison of mean Management Commitment scores by Roster Size categories; **B** Comparison of mean Supervisor Support scores by Roster Size categories; **C** Comparison of mean Management Commitment scores by Annual Call Volume categories; **D** Comparison of mean Supervisor Support scores by Annual Call Volume categories; **E** Comparison of mean Management Commitment scores by Population Served categories; **F** Comparison of mean Supervisor Support scores by Population Served categories

Pearson correlation matrices are presented by department level (Table 2) and individual level (Table 3). We observed a high positive correlation between annual call volume with roster size and with population served (Table 2). The relationship between these size variables and safety climate scores by organization type is presented in Fig. 2. We observed a high negative correlation between burnout and engagement on EMS runs (Table 2). We observed a moderate negative correlation between burnout on fire runs with engagement on fire runs. We observed a high positive correlation between job satisfaction and Management Commitment. A similar high positive correlation was observed between job satisfaction and Supervisor Support. At the individual level we observed similar patterns (Table 3). At the individual level we included the age and years of experience variables to our Pearson correlation matrix. We observed a high correlation between age and years of experience. In addition to the correlations present at the department level, among individuals we observed a moderate positive correlation between engagement on fire runs with Supervisor Support. Additionally, we observed a moderate positive correlation between safety compliance and Management Commitment.

**Table 2:** Department-level Pearson correlation coefficient matrix (*n* = 112)

	Percent EMS runs	Percent fire runs	Injury rate	Annual call volume	Roster size	Population served	Burnout on EMS runs	Engagement on EMS runs	Burnout on fire runs	Engagement on fire runs	Job satisfaction	Safety compliance	MC	SS
Percent EMS runs	1.00													
Percent fire runs	**− 0.87**	1.00												
Injury rate	0.25	− 0.26	1.00											
Annual call volume	0.17	− 0.13	0.37	1.00										
Roster size	0.13	− 0.11	0.31	**0.97**	1.00									
Population served	0.16	− 0.13	0.38	**0.98**	**0.96**	1.00								
Burnout on EMS runs	− 0.29	0.22	0.17	0.10	0.07	0.07	1.00							
Engagement on EMS runs	0.15	− 0.04	− 0.21	− 0.05	− 0.04	− 0.04	**− 0.70**	1.00						
Burnout on fire runs	− 0.26	0.21	0.02	0.05	0.05	0.03	**0.75**	− 0.44	1.00					
Engagement on fire runs	0.14	− 0.12	− 0.11	− 0.02	− 0.01	− 0.02	− 0.43	**0.51**	**− 0.65**	1.00				
Job satisfaction	− 0.10	0.19	− 0.10	0.03	0.03	0.03	− 0.44	0.46	− 0.45	0.42	1.00			
Safety compliance	0.17	− 0.18	− 0.10	0.03	0.05	0.04	− 0.44	0.28	− 0.49	0.37	0.41	1.00		
Management Commitment to safety (MC)	− 0.16	0.25	− 0.32	− 0.17	− 0.15	− 0.17	− 0.41	0.37	− 0.39	0.29	**0.78**	0.37	1.00	
Supervisor Support for safety (SS)	− 0.06	0.14	− 0.19	0.07	0.08	0.06	− 0.39	0.41	− 0.42	0.49	**0.76**	0.49	**0.58**	1.00

Bolded values indicate moderate (0.50–0.69) and high (0.70–0.99) correlations

**Table 3:** Individual-level Pearson correlation coefficient matrix (*n* = 8414)

	Age	Years of experience	Burnout on EMS runs	Engagement on EMS runs	Burnout on fire runs	Engagement on fire runs	Job satisfaction	Safety compliance	Management Commitment to safety	Supervisor Support for safety
Age	1.00									
Years of experience	**0.78**	1.00								
Burnout on EMS runs	0.15	0.11	1.00							
Engagement on EMS runs	− 0.12	− 0.23	**− 0.70**	1.00						
Burnout on fire runs	0.10	0.07	**0.70**	− 0.31	1.00					
Engagement on fire runs	− 0.18	− 0.26	− 0.28	0.24	**− 0.62**	1.00				
Job satisfaction	− 0.05	− 0.08	− 0.31	0.23	− 0.48	0.44	1.00			
Safety compliance	− 0.03	0.02	− 0.45	0.41	− 0.26	− 0.04	0.37	1.00		
Management Commitment to safety	− 0.16	− 0.01	− 0.49	0.33	− 0.41	0.15	**0.66**	**0.59**	1.00	
Supervisor Support for safety	− 0.19	− 0.20	− 0.26	0.22	− 0.45	**0.61**	**0.63**	0.21	0.41	1.00

Bolded values indicate moderate (0.50–0.69) and high (0.70–0.99) correlations

The results of the linear regression analyses are shown in Table 4. These analyses were used to estimate the relationship between safety climate scores and safety behaviors/organizational outcomes at the department level. Our findings indicate that all of our safety behavior/organizational outcomes are associated with both Management Commitment and Supervisor Support. Of note, job satisfaction was identified to have the strongest association between both Management Commitment and Supervisor Support as evidenced by the β of 0.79 and 0.67, respectively.

**Table 4:** Linear regression models examining the relationship between safety climate scores and safety behaviors/organizational outcomes

	Overall									
	Management Commitment^a^					Supervisor Support^a^				
	*n* = 118					*n* = 118				
	*B*	Standard error	95% CI	*β*	*p* value	*B*	Standard error	95% CI	*β*	*p* value
Safety behavior										
Safety Compliance	0.35	0.07	(0.22, 0.48)	0.55	**< 0.001**	0.68	0.11	(0.47, 0.89)	0.55	**< 0.001**
Organizational outcomes										
Burnout on EMS runs	− 0.26	0.06	(− 0.37, − 0.15)	− 0.49	**< 0.001**	− 0.41	0.10	(− 0.60, − 0.22)	− 0.39	**< 0.001**
Burnout on fire runs	− 0.17	0.04	(− 0.25, − 0.10)	− 0.47	**< 0.001**	− 0.31	0.06	(− 0.43, − 0.18)	− 0.43	**< 0.001**
Engagement on EMS runs	0.21	0.08	(0.05, 0.36)	0.30	**0.01**	0.41	0.15	(0.12, 0.71)	0.38	**0.01**
Engagement on fire runs	0.12	0.05	(0.03, 0.22)	0.28	**0.01**	0.42	0.07	(0.28, 0.56)	0.50	**< 0.001**
Job satisfaction	0.56	0.05	(0.46, 0.65)	0.79	**< 0.001**	0.91	0.09	(0.74, 1.09)	0.67	**< 0.001**

	Management Commitment to safet**y**														
	Career department^b^					Combination department^c^					Volunteer department^d^				
	*n* = 62					*n* = 33					*n* = 23				
	*B*	Standard error	95% CI	*β*	*p* value	*B*	Standard error	95% CI	*β*	*p* value	*B*	Standard error	95% CI	*β*	*p* value
Safety behavior															
Safety compliance	0.41	0.09	(0.23, 0.59)	0.53	**< 0.001**	0.24	0.13	(− 0.02, 0.50)	0.44	0.07	0.47	0.30	(− 0.18, 1.13)	0.40	0.14
Organizational outcomes															
Burnout on EMS Runs	− 0.23	0.07	(− 0.36, − 0.09)	− 0.40	**< 0.01**	− 0.22	0.11	(− 0.45, − 0.001)	− 0.43	0.05	− 0.26	0.36	(− 1.04, 0.52)	− 0.24	0.49
Burnout on fire runs	− 0.15	0.05	(− 0.25, − 0.05)	− 0.37	**< 0.01**	− 0.22	0.09	(− 0.41, − 0.03)	− 0.58	**0.03**	− 0.10	0.16	(− 0.44, 0.25)	− 0.14	0.55
Engagement on EMS runs	0.14	0.07	(− 0.002, 0.29)	0.26	0.05	0.37	0.13	(0.09, 0.64)	0.56	**0.01**	0.05	0.55	(− 1.15, 1.26)	0.03	0.93
Engagement on fire runs	0.12	0.06	(0.004, 0.25)	0.28	**0.04**	0.25	0.09	(0.07, 0.43)	0.63	**0.01**	0.28	0.26	(− 0.30, 0.85)	0.27	0.32
Job satisfaction	0.56	0.08	(0.41, 0.72)	0.71	**< 0.001**	0.63	0.11	(0.39, 0.87)	0.90	**< 0.001**	0.60	0.18	(0.21, 0.99)	0.69	**0.01**

	Supervisor Support for safety														
	Career department^b^					Combination Department^c^					Volunteer department^d^				
	*n* = 62					*n* = 33					*n* = 23				
	*B*	Standard error	95% CI	*β*	*p* value	*B*	Standard error	95% CI	*β*	*p* value	*B*	Standard error	95% CI	*β*	*p* value
Safety behavior															
Safety compliance	0.64	0.20	(0.23, 1.04)	0.40	**< 0.01**	0.58	0.13	(0.30, 0.85)	0.62	**< 0.01**	0.80	0.26	(0.24, 1.36)	0.62	**0.01**
Organizational outcomes															
Burnout on EMS runs	− 0.47	0.14	(− 0.75, − 0.20)	− 0.42	**< 0.01**	− 0.45	0.12	(− 0.70, − 0.20)	− 0.51	**< 0.01**	− 0.05	0.38	(− 0.89, 0.78)	− 0.04	0.90
Burnout on fire runs	− 0.37	0.10	(− 0.57, − 0.18)	− 0.47	**< 0.01**	− 0.32	0.12	(− 0.57, − 0.08)	− 0.49	**0.01**	− 0.12	0.16	(− 0.48, 0.23)	− 0.17	0.47
Engagement on EMS runs	0.38	0.15	(0.08, 0.67)	0.35	**0.01**	0.74	0.13	(0.48, 1.01)	0.67	**< 0.001**	− 0.11	0.58	(− 1.37, 1.15)	− 0.05	0.85
Engagement on fire runs	0.49	0.11	(0.27, 0.71)	0.54	**< 0.001**	0.47	0.09	(0.28, 0.65)	0.69	**< 0.001**	0.38	0.27	(− 0.21, 0.96)	0.34	0.19
Job satisfaction	1.06	0.17	(0.71, 1.40)	0.66	**< 0.001**	0.87	0.14	(0.59, 1.16)	0.73	**< 0.001**	0.81	0.11	(0.57, 1.06)	0.86	**< 0.001**

*n*'s do not add up to 125 due to missing values (n = 7) for adjusted covariates

Bolded values are statistically significant at an alpha level of 0.05

^a^Adjusted for roster size (10–29, 30–50, 51–101, 102–2303), annual call volume (75–821, 822–3288, 3289–9080, 9081–451,069), population served (590–9999, 10,000–26,999, 27,000–80,000, 80,000–2,500,000)

^b^Adjusted for roster size (11–44, 45–80, 81–151, 152–2303), annual call volume (1150–3199, 3200–7379, 7380–14,499, 15,000–451,069), and population served (1000–24,999, 25,000–57,030, 57,031–129,999, 130,000–2,500,000)

^c^Adjusted for roster size (10–24, 25–40, 41–63, 64–361), annual call volume (240–856, 857–2495, 2496–5605, 5606–40,847), and population served (1400–8099, 81,000–20,499, 20,500–35,999, 36,000–452,000)

^d^Adjusted for roster size (16–23, 24–29, 30–41, 42–78), annual call volume (75–119, 120–273, 274–594, 595–896), and population served (590–2167, 2468–4999, 5000–14,999, 15,000–27,000)

The results of the linear regression analyses stratified by organization type are shown in Table 4. These analyses were used to estimate the relationship between safety climate scores and safety behaviors/organizational outcomes at the department level stratified by organization type. Job satisfaction was the only outcome for which we observed an association across all organization types. This was observed for both Management Commitment and Supervisor Support. Our findings indicate that all of our safety behavior/organizational outcomes are associated with Supervisor Support for career and combination departments, but not with Management Commitment. Overall, we did not observe the same associations between safety behavior and organizational outcomes (excluding job satisfaction) with safety climate scores present among volunteer departments. We did observe a positive association between safety compliance and Supervisor Support in volunteer departments for the fully adjusted models.

Examination of individual-level descriptive characteristics shown was used to evaluate the relationship between injury status and safety climate scores (Table 5). Among all individuals, for a one-unit increase in Management Commitment the odds of injury decreases by 3% (OR 0.97, 95% CI 0.97–0.98). Among all individuals, for a one-unit increase in Supervisor Support the odds of injury decreases by 4% (OR 0.96, 95% CI 0.94–0.98). A similar association was observed among career fire department members. Among combination fire department members, for a one-unit increase in Management Commitment the odds of injury decreases by 3% (OR 0.97, 95% CI 0.95–0.99). We did not observe an association with injury status and Supervisor Support in combination or volunteer departments. Additionally, we did not observe an association with injury status and Management Commitment for volunteer departments.

**Table 5:** Multilevel logistic regression between safety climate and injury status among individuals stratified by organization type

	All individuals (*n* = 7689), stations (*n* = 609), departments (*n* = 125)			Individuals in career departments (*n* = 6334), stations (*n* = 490), departments (*n* = 67)			Individuals in combination departments (*n* = 1021), stations (*n* = 87), departments (*n* = 34)			Individuals in volunteer departments (*n* = 334), stations (*n* = 32), departments (*n* = 24)		
	Estimate	95% CI	OR (95% CI)	Estimate	95% CI	OR (95% CI)	Estimate	95% CI	OR (95% CI)	Estimate	95% CI	OR (95% CI)
Unadjusted												
Management Commitment	− 0.03	(− 0.04, − 0.02)	0.97 (0.96, 0.98)	− 0.02	(− 0.03, − 0.01)	0.98 (0.97, 0.99)	− 0.03	(− 0.06, − 0.01)	0.97 (0.94, 0.99)	0.06	(− 0.05, 0.16)	1.06 (0.95, 1.17)
Supervisor Support	− 0.05	(− 0.07, − 0.03)	0.95 (0.93, 0.97)	− 0.06	(− 0.08, − 0.03)	0.94 (0.92, 0.97)	− 0.02	(− 0.07, 0.03)	0.98 (0.93, 0.97)	0.02	(− 0.09, 0.13)	1.02 (0.92, 1.14)
Adjusted^a^												
Management Commitment	− 0.03	(− 0.03, − 0.02)	0.97 (0.97, 0.98)	− 0.02	(− 0.03, − 0.01)	0.98 (0.97, 0.99)	− 0.03	(− 0.06, − 0.01)	0.97 (0.95, 0.99)	0.06	(− 0.05, 0.17)	1.06 (0.95, 1.18)
Supervisor Support	− 0.04	(− 0.06, − 0.02)	0.96 (0.94, 0.98)	− 0.05	(− 0.07, − 0.02)	0.96 (0.93, 0.98)	0.01	(− 0.05, 0.06)	0.99 (0.95, 1.06)	0.02	(− 0.09, 0.13)	1.02 (0.91, 1.14)

^a^Adjusted for age, years of experience, sex (male, female), and officer status (non-officer, officer, leadership)

## Discussion

For our study we evaluated and reported on the descriptive characteristics of the FOCUS beta-test survey sample at the individual and department level, stratified by fire department organization type. Additionally, we examined the association between safety behaviors, organizational outcomes, and safety outcomes with safety climate scores. Overall, we observed a 10-point difference between Management Commitment and Supervisor Support when comparing the overall department mean scores. Additionally, we observed a reduction in the odds of self-reported injury for a one-unit increase in Management Commitment and Supervisor Support among our population.

### Management Commitment and Supervisor Support

Management Commitment was the lower scoring safety climate score. When we stratified Management Commitment by organization type, we observed a dose–response relationship, which indicated that the more career a department became the lower the Management Commitment score became. We hypothesize that this means that leaders in more career departments may have work to do to better communicate their actual support for safety among their members. Prior research in the trucking industry observed a protective impact of safety climate on safety behavior and injury that was moderated by the quality of safety communication by supervisors (Huang et al. 2018). Future research should further investigate this dose–response finding and see what is changing in terms of the organizational environment that may lead to these reductions in perceived Management Commitment. Those factors may then become targets for intervention. We did not observe the same dose–response when we stratified Supervisor Support by organization type.

### Management Commitment and department size

Among our sample, career fire departments were typically larger in roster size, annual call volume, and population served compared to combination or volunteer departments. Due to the larger roster size, we believe that rank-and-file members within career departments are less likely to interact with leadership at the management level. This may be what is driving the differences observed when stratified by organization type for Management Commitment. This finding suggests to us that management of career departments, specifically, need to improve their interaction at the station level. An effort by management to visit members at the stations may be warranted to increase the perception of Management Commitment and be more reflective of the Supervisor Support scores we observed. The executive leadership walkaround exemplifies this idea and has been used successfully in the healthcare industry (Schwendimann et al. 2013; Sexton et al. 2018); however, we do not believe that this intervention has been evaluated specifically in the fire service.

### Supervisor Support and department size

We observed that combination fire departments look more like career because they have lower Supervisor Support for safety scores compared to volunteer. Since this score is directly linked to the interaction between officers and non-officer members at the station level, this is something that should be addressed by career and combination departments to ensure that their rank-and-file feel supported by their direct supervisors.

### Safety climate and job satisfaction

We observed a positive association of both Management Commitment and Supervisor Support with job satisfaction across all organization types. The association was strongest with Supervisor Support. These findings held for the fully adjusted models and support the findings from our correlation matrices. Future work should examine Management Commitment interventions to further increase the job satisfaction score.

### Safety climate and organizational outcomes

Across career and combination departments we observed that safety climate is positively associated with safety behavior and organizational outcomes that are reflective of employee well-being. These associations were notably stronger for Supervisor Support when compared to Management Commitment for career and combination departments. Additionally, across career, combination, and volunteer departments we observed a positive association between safety compliance and Supervisor Support. Connecting this to our conceptual framework (Taylor et al. 2019), this further supports the idea that supervisors matter and may play an important role in preventing injuries.

### Safety climate and injury

Overall and for career members, we observed that the individuals had lower odds of self-reported injury in the past 12 months for every one-unit increase of Management Commitment and of Supervisor Support. There was a borderline association observed between injury status and Supervisor Support among members in combination departments. Our lack of associations among members at volunteer departments may be due to the small sample size for this organization type. We recommend that these results be interpreted cautiously. Overall, this finding suggests that Supervisor Support is a slightly stronger driver in lowering the odds of injury than Management Commitment, especially in combination departments. Future research should examine this relationship with a larger volunteer sample to better understand the true relationship in this organization type.

### Relevance

Previous research has been conducted to evaluate different aspects of safety climate as it relates to the physical and mental health of firefighters (Armstrong et al. 2016). Additional work has been conducted to investigate the effect of burnout on firefighters (Smith et al. 2020; Jeung and Chang 2021). Reducing burnout, among members of the fire service, may aid in injury prevention. Further understanding of safety climate within the US fire service is necessary in order to provide physical and mental health prevention among the workers in this occupational group.

Observed gaps in terms of the relationship between safety climate and organizational outcomes across career, combination, and volunteer departments may indicate the heterogeneity in self-determination and work attitude (Deci and Ryan 2012) across career and volunteer firefighters. Volunteer firefighters are more likely to be intrinsically motivated and work in a highly autonomous way. Accordingly, they are more likely to find task identity and significance from their own work behaviors, rather than relying on external feedback from management or supervisors (Gagné et al. 1997). Moreover, weaker relationships between safety climate and injury status in combination and volunteer departments than in career departments suggest that a stronger sense of independence and autonomy among volunteer firefighters can hamper the effective translation of safety climate into individual-level safety behaviors and outcomes. Considered jointly, safety climate facilitation efforts need to properly consider the unique work attitudes of volunteer firefighters for the optimal workplace safety, health, and well-being promotion.

Our prior work (Taylor et al. 2019) is supported by our current findings which provides more insight into the relationships between organizational outcomes and safety climate scores among the FOCUS beta-test respondents, specifically in understanding the differences across organization types.

### Strengths

Our study had important strengths. The FOCUS beta-test is comprised of a large sample size. Participating fire departments were selected using a geographically stratified random sampling method, which reduces concerns for selection bias based on sampling methods This method of sampling is appropriate for obtaining representation across the US. Another strength of the FOCUS survey is that the same individual reported on their individual perception to burnout and engagement on EMS runs and on fire runs. This survey design controls for confounding since the same person is answering for both EMS and fire runs. We observed that reported burnout on EMS runs was higher, while engagement was lower in response to EMS runs, meaning that the individual going on the EMS runs experiences more burnout and feel less engagement. These findings are consistent with the increased percentage of EMS runs compared to fire runs observed in our sample.

To our knowledge, we are the first research group to evaluate safety climate in a sample that includes career, combination, and volunteer fire departments. This is important for our work on safety climate as it provides a greater understanding of the importance of investigating safety by organization type.

### Limitations

There are several limitations to the current study. Within our sample we had an over-representation of career versus volunteer firefighters. This oversampling of career departments is particularly evident when looking at our analyses stratified by organization type. We observed limited associations among volunteer departments, which may be driven by the small number of volunteer departments in our sample. In future analyses, if possible, a larger sample size of volunteer fire departments is warranted to examine whether there are differences in associations in comparison with our findings. While our method reduced concerns of selection bias by sampling, it is possible that selection bias could still be present because we had an average 66% response rate at the department level.

Another limitation is that injury status was only captured at the individual level through self-report recalling injuries that occurred over the past 12 months. There is a possibility that minor injuries were not reported due to recall bias depending on when the injury occurred and when the respondent participated in our survey. Since injury was only evaluated on the individual level, the department-level mean Management Commitment and Supervisor Support scores were attributed to all individuals in the corresponding department. Thus, there is potential ecological fallacy present, which would result in a skewing of the true association. Additionally, there were a limited number of individuals that reported having an injury in the 12 months prior to completing the FOCUS survey, especially in volunteer fire departments. Thus, cautious interpretation of the logistic regression findings is warranted.

## Conclusions

Our research evaluated the results from 125 fire departments that completed the FOCUS beta-test survey. Our results indicate that safety climate is positively associated with safety behavior, safety outcomes, and organizational outcomes reflecting employee well-being. There is a notable dose–response in that as a department becomes more career its Management Commitment to Safety decreases. And while we have controlled for roster size, annual call volume, and population served, our results indicate that it is not just being busy, but something else within the organization that contributes to this marked difference. Future studies should elucidate the phenomena of how Management Commitment declines as a department becomes more career-like.

## Data Availability

The datasets generated and analyzed during the current study are not publicly available due to conditions of the IRB but are available from the corresponding author on reasonable request.

## Abbreviations

FOCUS: Fire Service Organizational Culture of Safety survey
US: United States
EMS: Emergency medical services
FEMA: Federal Emergency Management Agency
SD: Standard deviation
EMT: Emergency medical technician
CPSE: Center for Public Safety Excellence
ISO: Insurance Services Office
OR: Odds ratio
CI: Confidence interval
